
JOURNAL INFORMATION
==============================
NLM Title Abbreviation: South Med Rec
NLM Journal ID: 0027317

The Southern Medical Record

Publisher: Biomedical Journal Digitization Project

ARTICLE INFORMATION
==============================
PMCID: PMC9071657
PMID: 36021687
Article version: 1
Subjects: Editorial and Miscellaneous

Hydrate of Chloral

Print publication date: 1880 May 20
Volume: 10
Issue: 5
First page: 192
Last page: 192
License: This work is free of known copyright restrictions. For more information, please see PMC Back Issue Digitization.
License URL: https://creativecommons.org/publicdomain/mark/1.0/

==============================

### *HYDRATE OF CHLORAL.*

Dr. H. H. Kane, 191 West 10th street, New York, specially requests members of the profession with any experience in the use of hydrate of chloral to answer the following questions, and give any information they may possess with reference to the literature of the subject:

1. What is your usual commencing dose?
2. What is the largest amount you have administered at one dose, and the largest amount in twenty-four hours?
3. In what diseases have you used it (by the mouth, rectum, or hypodermically), and with what results?
4. Have you known it to affect the sight?
5. Have you ever seen cutaneous eruptions produced by it?
6. Have you known it to affect the sexual organs? If so, how?
7. Do you know of any instances where death resulted from or was attributed to its use? If so, please give full particulars as to disease for which given; condition of pulse, pupils, respiration and *temperature;* manner of death; condition of heart, lungs and kidneys; general condition, age, temperament, employment, etc., etc., etc. If an autopsy was held, please state the condition there found.
8. Have you seen any peculiar manifestations from chloral—as tetanus, convulsions, or delirium?
9. Do you know of any cases of the chloral habit? If so, please state the amount used, the disease for which the drug was originally administered, the person's temperament, and the present condition of the patient, with reference to the state of body and mind in general, and the various organs and systems in particular.

---

### SPECIAL NOTICES.

WM. R. WARNER & CO.—We desire to call the attention of our readers to the advertisement of this house. It is one of the most reliable houses in the United States, and all the preparations which they advertise can be depended upon to be as represented. We have used their medicines ourselves, and have never been disappointed.

It is becoming more and more necessary to make remedies as little repulsive to patients as possible, and therefore those elegant preparations, as are many of the elixirs, syrups, and sugar-coated pills, are becoming popular among physicians; but a frequent drawback is the unreliability of the preparations of many manufactories. This objection, however, we know, does not hold in regard to those of Warner & Co.—*Cincinnati Medical News.*

We would call attention to the advertisement, on page 9, of **Messrs. HENRY THAYER & CO.**

This is an old and honorable house, having been established in their branch of Pharmaceutical Chemistry over thirty years.

All of their preparations are faithfully made of full strength, elegant in appearance, and have deservedly won a wide reputation. Physicians and druggists may depend upon the correctness and accuracy of any preparation bearng their label.

Their list of new Fluid Extracts is extensive, and the genuineness of indigenous raw material is certified at the Botanical Gardens of Harvard University.

THE NAME **PARKE, DAVIS & CO.** on the label of a package has become a guarantee to the physicians of this country, of honesty in the manufacture of the preparation. The firm is reaping the fruits which such a reputation naturally brings, and stands to-day second to no firm of pharmacists in this country in the extent of its business.

The Profession has, moreover, been placed under obligations to it for the great majority of the valuable New Remedies which have during the past few years been added to the materia medica. Inasmuch as it makes a specialty of New Remedies, physicians will do well in testing these to secure PARKE, DAVIS & Co's preparations of them before passing judgment on their merits.

The later additions which Parke, Davis & Co. have introduced are **Jamaica Dog Wood,** a substitute for opium; **Manaca,** the Brazilian antirheumatic remedy; and **Ergotæ Purificatus,** a constant preparation of ergot and one peculiarly adapted for hypodermic administration.

EXTRACT OF A LETTER FROM DR. HUNTER McGUIRE.

*Richmond, Va., April* 1880. * * * * * In a host of diseases where a powerful tonic and alterative is wanted, the BEDFORD ALUM AND IRON MASS AND WATER is of inestimable value. HUNTER McGUIRE.
